# Dataset N-doping of alumina thin film support of catalysts

**DOI:** 10.1016/j.dib.2021.107383

**Published:** 2021-09-20

**Authors:** Aleksey M. Dmitrachkov, Ren I. Kvon, Anna V. Nartova

**Affiliations:** Boreskov Institute of Catalysis SB RAS, Lavrentieva Ave., 5, Novosibirsk 630090, Russia

**Keywords:** N-doped catalyst support, Model alumina, XPS, STM

## Abstract

The search for the ways of thermal stabilization of supported metal catalysts is an important challenge in the modern catalysis. Chemical modification of support seems to be the most versatile approach to stabilize the metal particles against sintering and alter their catalytic performance. Also for such modification nitrogen doping can be used and is considered rather perspective. In a recent manuscript (A.M. Dmitrachkov, R.I. Kvon, A.V. Nartova, N-doping of alumina thin film support to improve the thermal stability of catalysts: preparation and investigation, Appl. Surf. Sci.) we have developed the procedure of N-doping of alumina thin film grown at the surface of metal substrate. Proposed N-doped model alumina support is suitable for catalysis – oriented surface science studies and improves the resistance of supported metal particles against thermal driven sintering. Herein, we provide useful complementary data for the characterization of the prepared materials in the form of:

in situ / ex situ XPS (X-ray photoelectron spectroscopy) spectra at every stage of sample preparation, including angle resolved XPS experiments and thermal stability tests;

STM (scanning tunneling microscopy) images of supported gold catalysts. Presented data support the proposed mechanism of film formation and modification.

**Specifications Table**

**Table:** 

Subject	Materials Science: Surfaces, Coatings and Films
Specific subject area	Inorganic Chemistry and Physical Characterization
Type of data	Image Figure
How data were acquired	X-ray photoelectron spectra were acquired on a VG ESCALAB HP electron spectrometer (basic chamber pressure was ∼10^−9^ mbar, Bayard-Alpert Type Ionization Vacuum Gauges). Scanning tunneling microscopy images were recorded on an UHV (ultrahigh-vacuum) variable temperature STM VT-7000 (RHK Technology, USA) (basic chamber pressure was ∼10^−10^ mbar, Gamma Vacuum Ion pump).
Data format	Raw Analyzed
Parameters for data collection	XPS source – nonmonochromatic Mg Kα (hν=1253.6eV) X-Ray gun (XR-3 source by VG Scientific) at ∼150 W power (anode voltage of 10 kV and emission current of 15 mA); Hemispheric Analyzer – Constant Analyzer Energy (Fixed Analyzer Transmission) mode with retard grid in front of entrance analyzer slit (i.e., transmission function is constant). Pass Energy = 50 eV (Ag3d peak fwhm = 1.75 eV) used for survey spectra and Pass Energy = 20 eV (Ag3d peak fwhm = 1.34 eV) was chosen for high - resolution spectra; typical angle of analysis (θ) was 30° to the normal to the sample plane; angle resolved measurements were done in θ range of 30° – 85°. STM - the cut Pt/Ir tips, topography and tunneling current images were acquired simultaneously. Typical tunneling current of 0.5 nA at voltage in range of ±200 mV to ±2200 mV.
Description of data collection	XPS - The foil specimens were mounted on spectrometer sample holder by tungsten wire legs spot-welded to the backside of the disks to provide resistive heating to anneal the samples. The sample temperature was measured by K type thermocouple spot-welded directly to the back of samples. The flows of gases were tuned up in by-pass line by leak valves and then switched directly into XPS analytic chamber to carry out *in situ* XPS experiments. STM – samples were placed on standard sample holder of ST - microscope VT-7000 (RHK Technology, USA), contacts were spot-welded directly to the sample.
Data source location	Boreskov institute of catalysts Siberian Branch of Russian Academy of Science (BIC SB RAS), Novosibirsk, Russia.
Data accessibility	https://data.mendeley.com/datasets/m7sgtyhh5w/draft?a=24aa0e15-bdb8-491f-8d5f-98add5c78a2f (https://doi.org/10.17632/m7sgtyhh5w.1)
Related research article	A.M. Dmitrachkov, R.I. Kvon, A.V. Nartova, N–doping of alumina thin film support to improve the thermal stability of catalysts: preparation and investigation, Applied Surface Science 566 (2021) 150631 https://doi.org/10.1016/j.apsusc.2021.150631

**Value of the Data**

• These data are important for the substantiation of the mechanisms proposed for the formation and N – doping of alumina film on the surface of metal substrate.
• Researchers in surface and materials science and catalysis can find the spectroscopy data, useful for characterizing analogous materials and application of such materials for catalyst designing.
• Reported data can be used for the design of new catalysts based on N-doped alumina supports.

## 1 Data Description

The data in this article refer to the samples described in “N-doping of alumina thin film support to improve the thermal stability of catalysts: preparation and investigation” [1] and in “Nitrogen-doped alumina carrier for sintering resistant gold supported catalysts” [2].

**Fig. 1:**
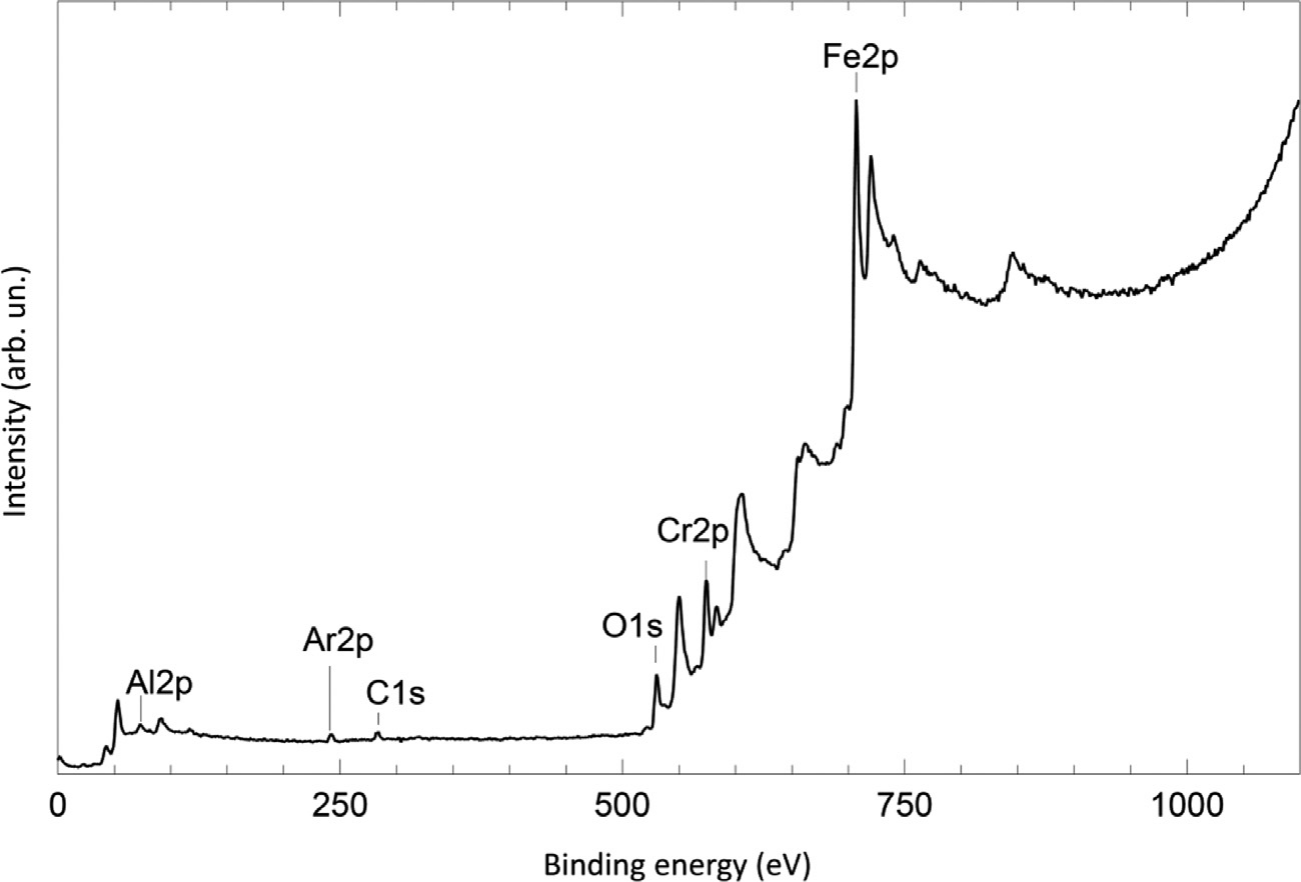
Survey spectrum of Ar^+^ etched FeCrAl foil (**5_FCA-N_0**).

**Fig. 2:**
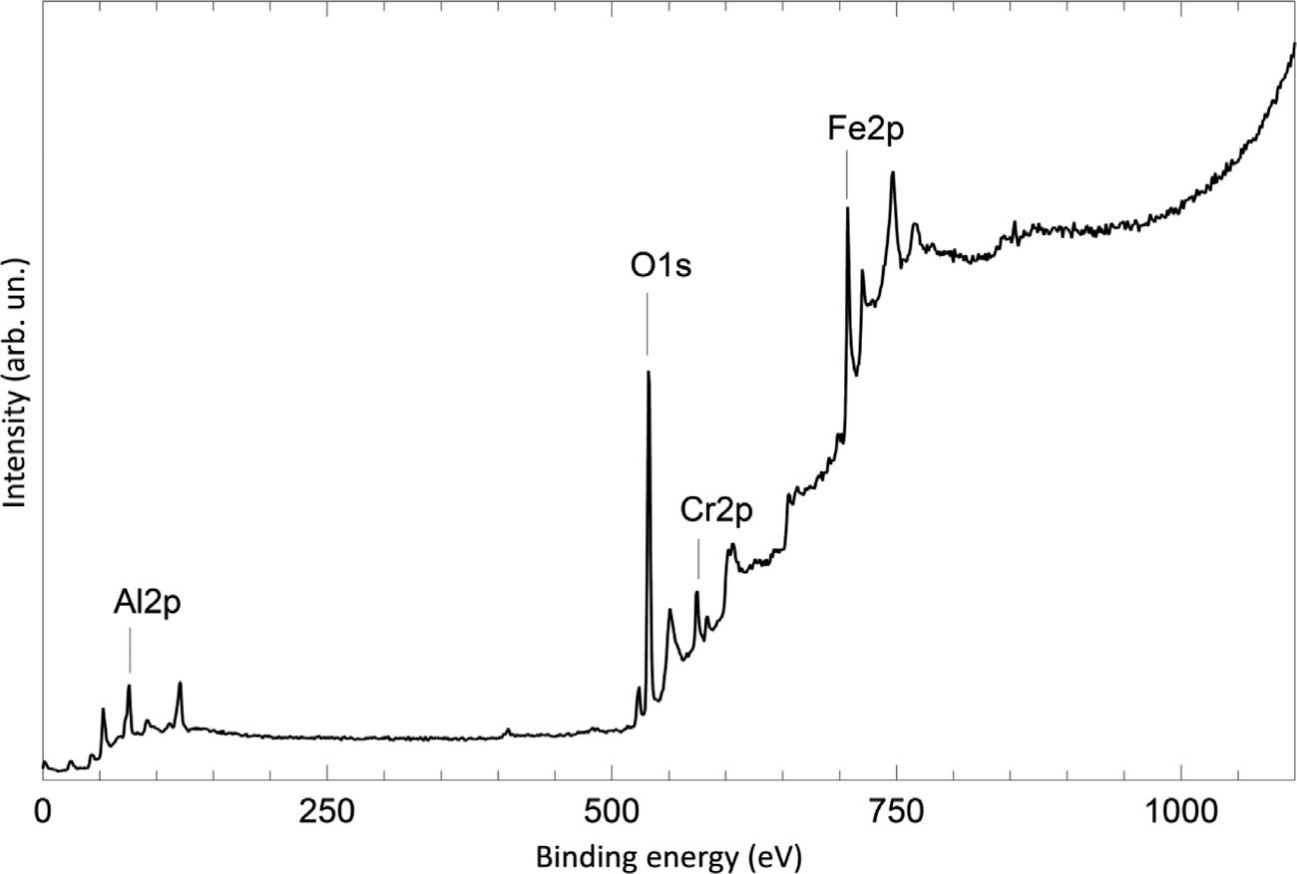
Survey spectrum of sample with non-modified AlOx film over FeCrAl substrate (**0**_**FCA_1**).

**Fig. 3:**
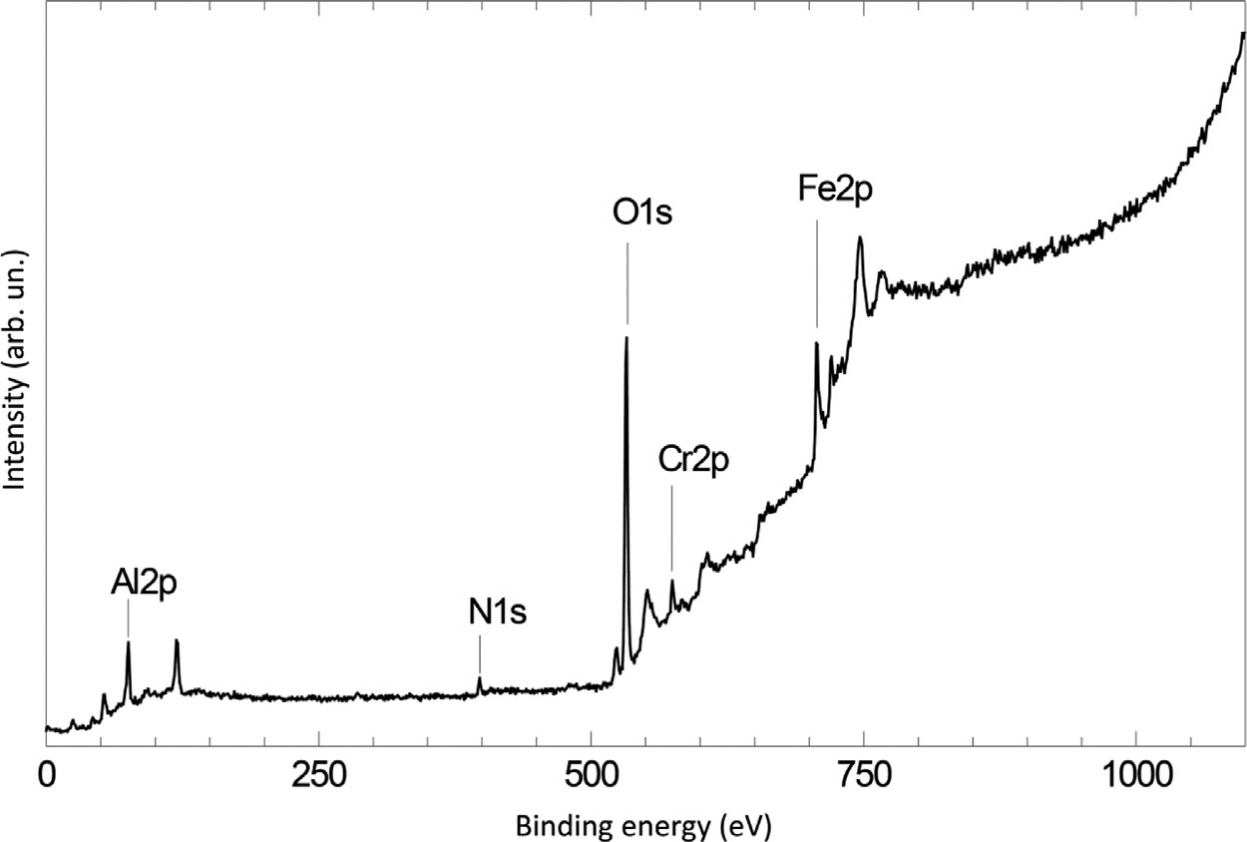
Survey spectrum of sample with modified AlOx-N film over FeCrAl substrate (**5_FCA-N_1**).

**Fig. 4:**
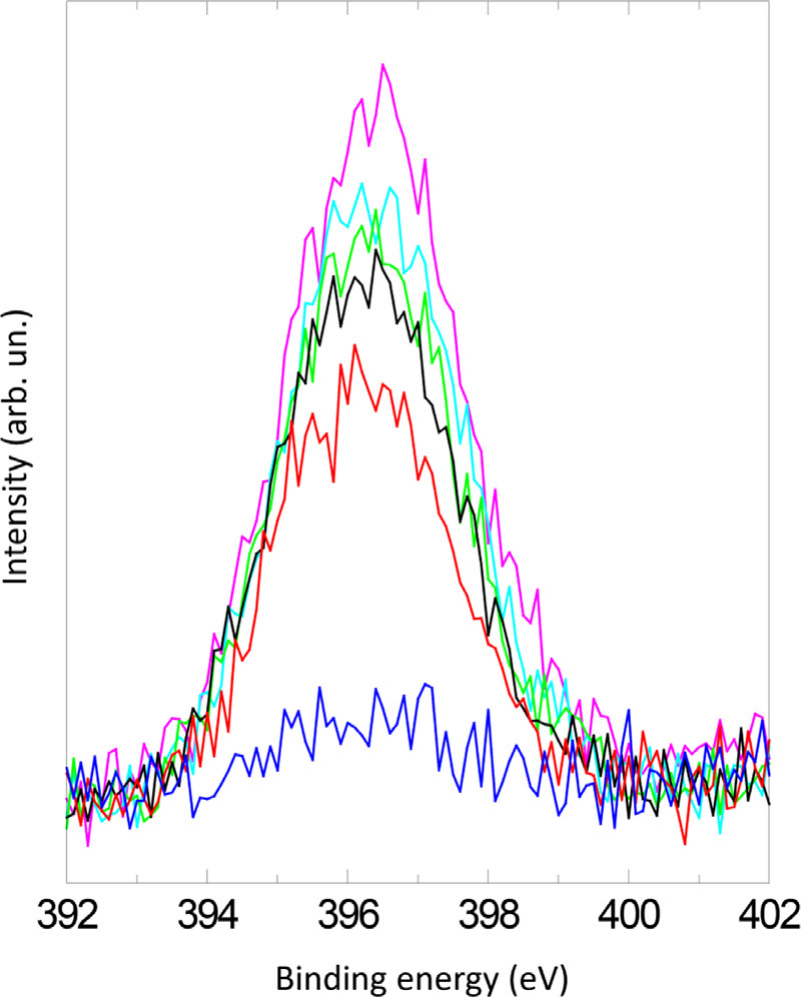
Evolution of N1s line during NO treatment (7 × 10^−7^ mbar NO exposition at 670°C) (**6_FCA-N_1**).

**Fig. 5:**
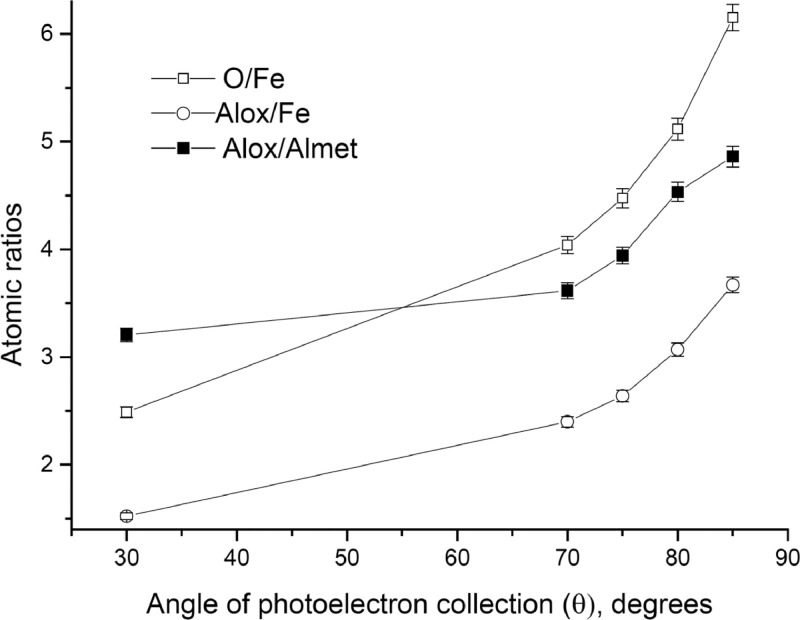
Angular dependence of O/Fe, Alox/Fe and Alox/Almet atomic ratios (sample # 5, **5_FCA-N_1**).

**Fig. 6:**
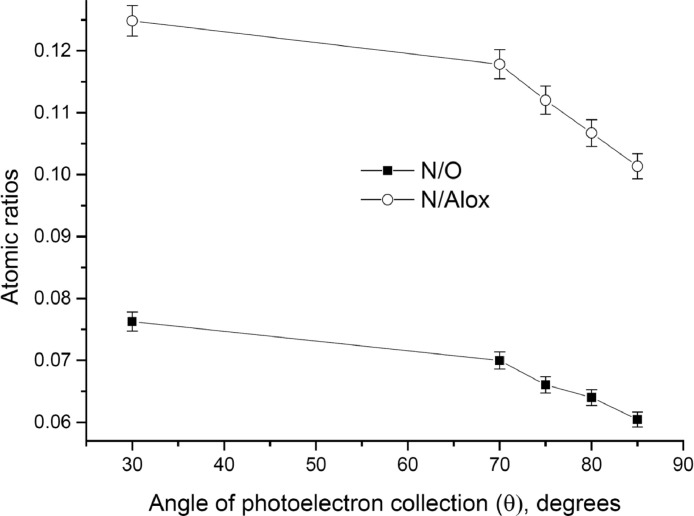
Angular dependence of N/O and N/Alox atomic ratios (sample # 5, **5_FCA-N_1**).

**Fig. 7:**
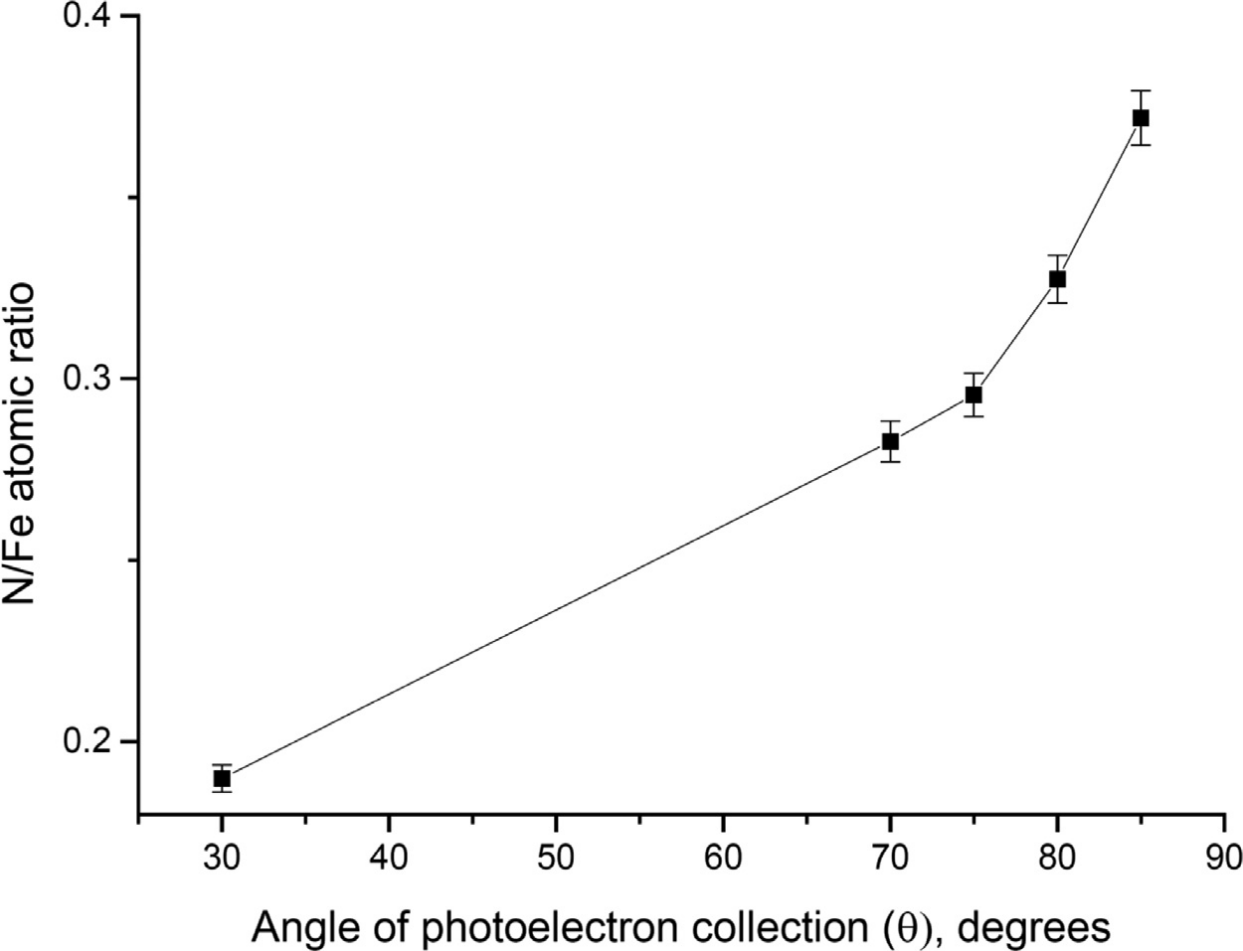
Angular dependence of N/Fe atomic ratio (sample # 5, **5_FCA-N_1**).

**Fig. 8:**
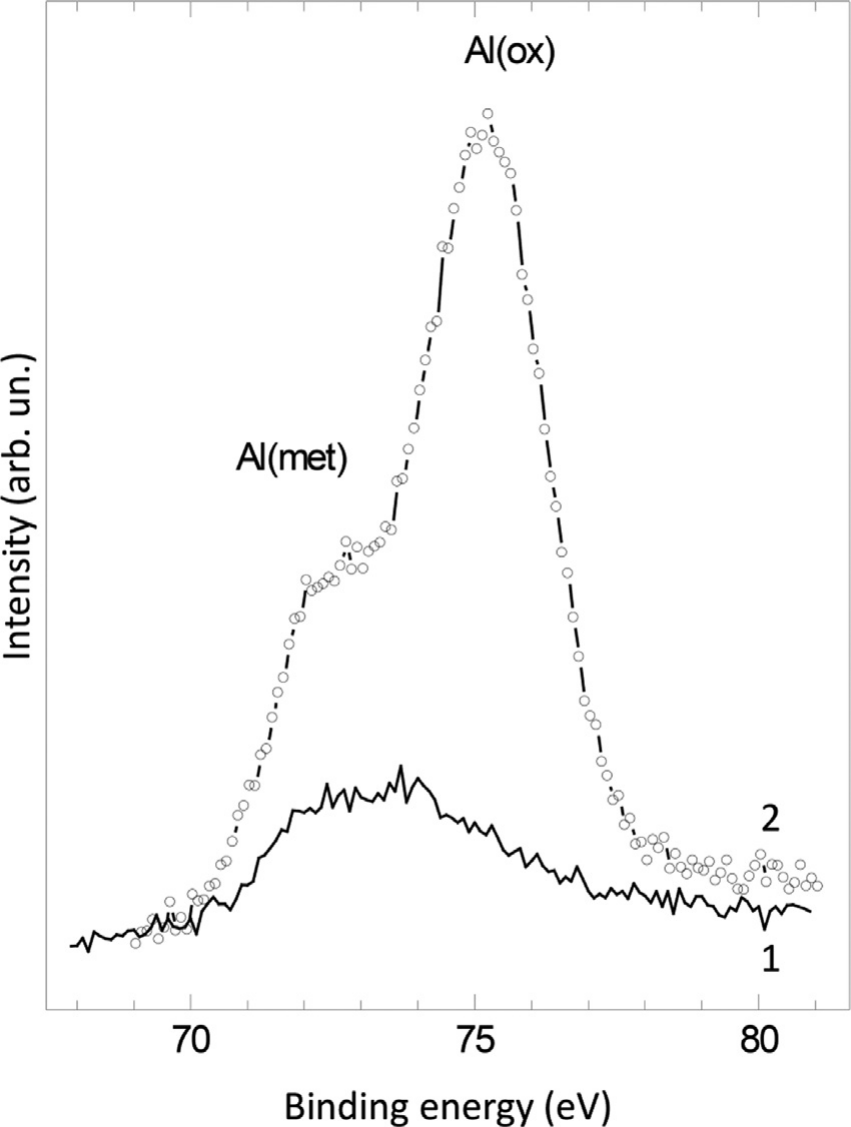
XPS spectra of Al2p line before (1) (**3_FCA-N_0**) and after (2) (**3_FCA-N_1**) N – doped film formation.

**Fig. 9:**
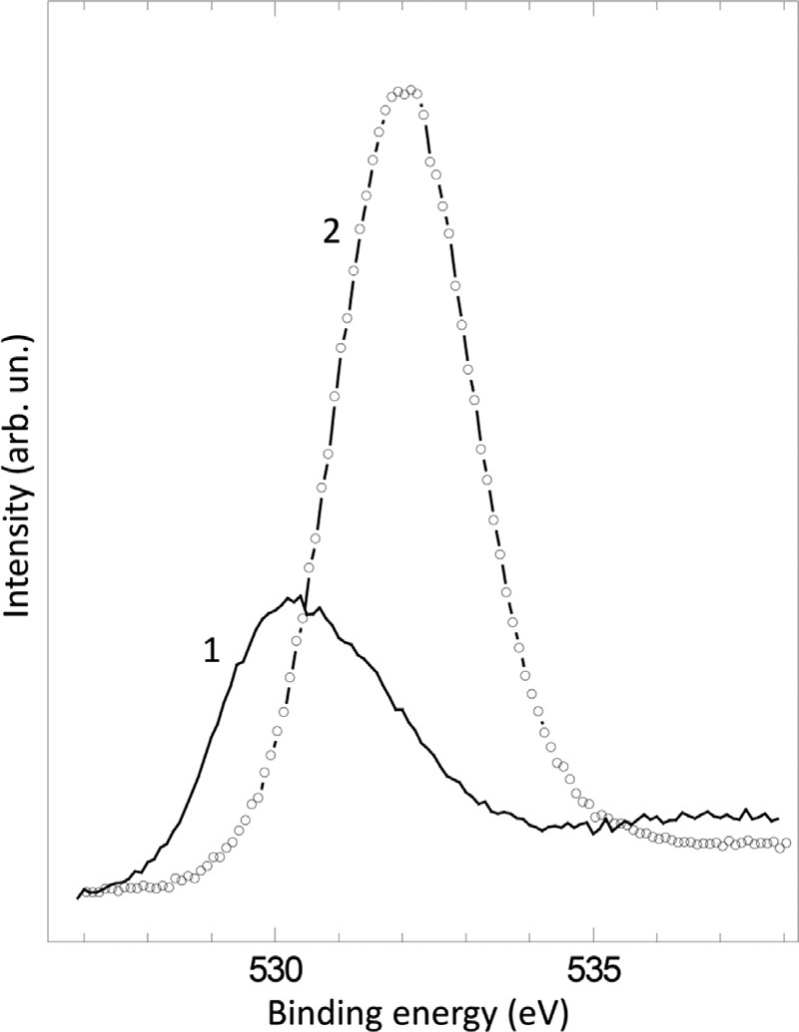
XPS spectra of O1s line before (1) (**3_FCA-N_0**) and after (2) (**3_FCA-N_1**) N – doped film formation.

**Fig. 10:**
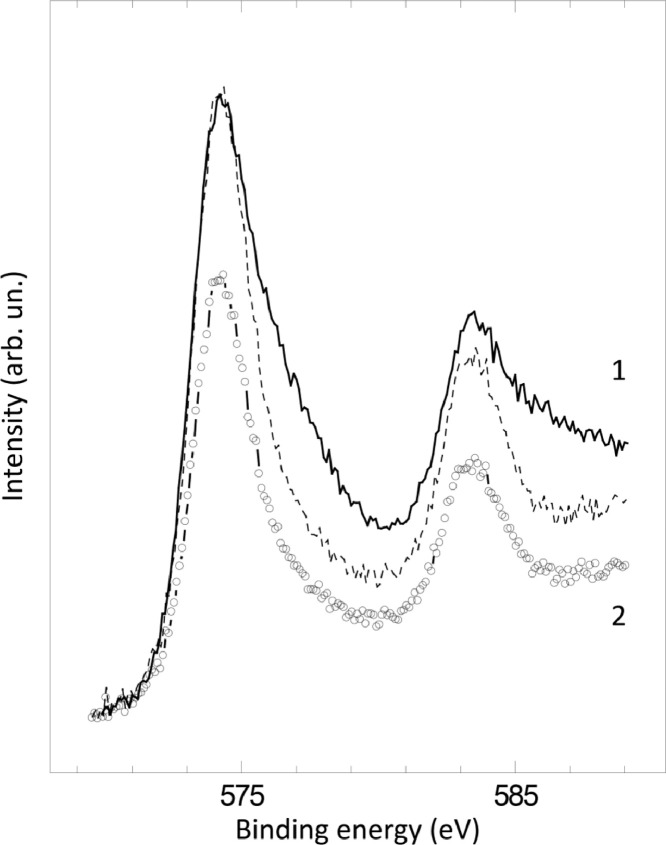
XPS spectra of Cr2p line before (1) (**3_FCA-N_0**) and after (2) (**3_FCA-N_1**) N – doped film formation (dashed line is (2) spectrum normalized to the intensity of (1) spectra for the comparison purposes).

**Fig. 11:**
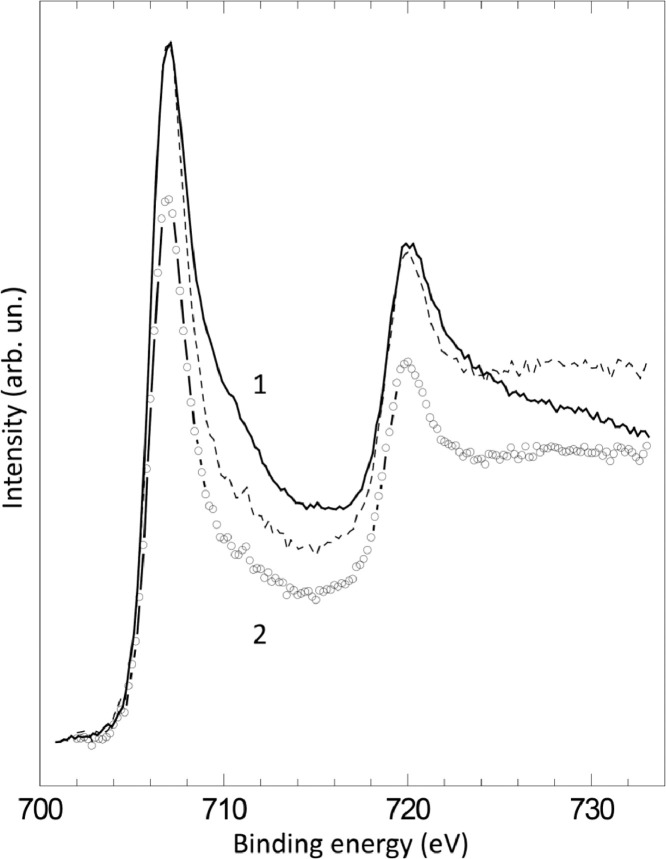
XPS spectra of Fe2p line before (1) (**3_FCA-N_0**) and after (2) (**3_FCA-N_1**) N – doped film formation (dashed line is (2) spectrum normalized to the intensity of (1) spectra for the comparison purposes).

**Fig. 12:**
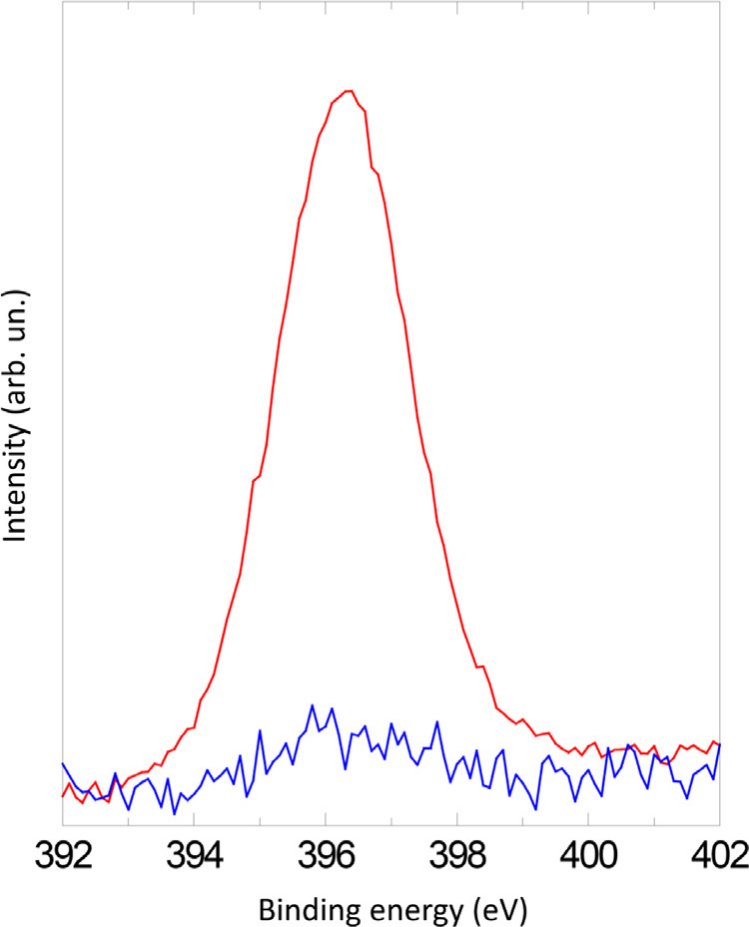
XPS spectra of N1s line before (blue) and after (red) NO treatment (**6_FCA-N_1**, 7 × 10^−7^ mbar), when NO presents in residual vacuum.

**Fig. 13:**
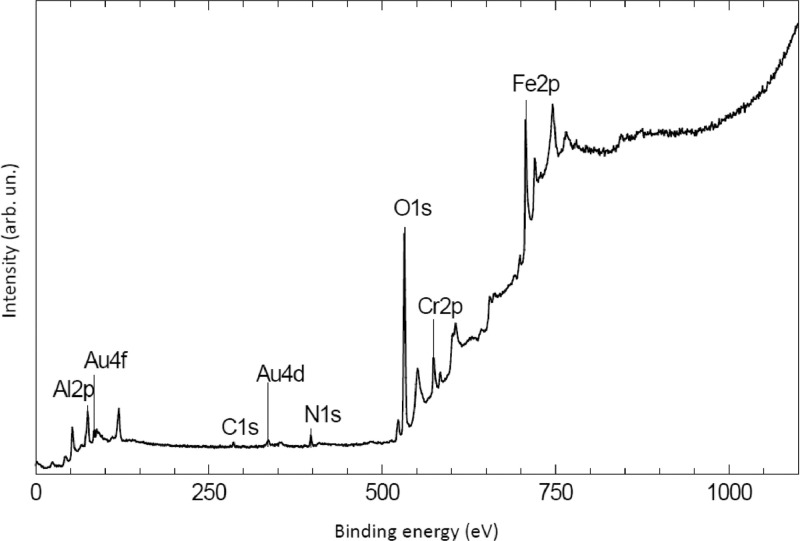
Survey spectrum of sample Au on N-doped AlOx/FeCrAl (**Au_FCA-N**).

**Fig. 14:**
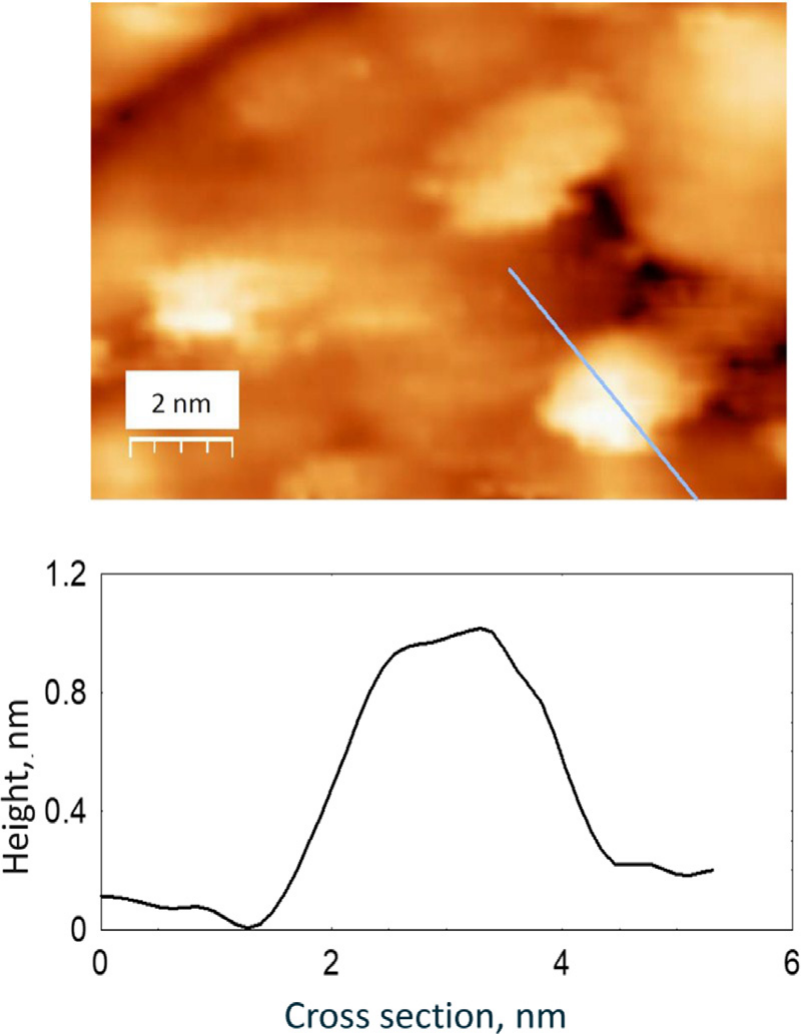
STM image of model catalyst Au/AlOx/FCA (13.4 nm × 8.5 nm) (**Au_FCA**) and cross section of the surface with gold particle.

**Fig. 15:**
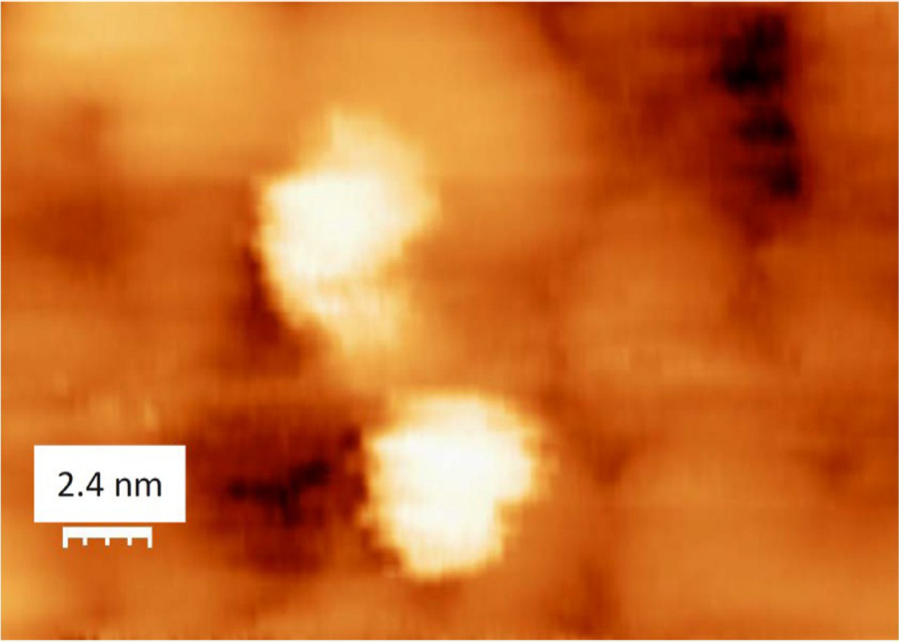
STM images of model catalyst Au/N-AlOx/FCA (24.0 nm × 14.6 nm) (**Au_FCA-N**).

The survey XPS spectra show presence of main components of foil used as substrate for sample preparation (Fe, Cr, Al, O, Ar) after foil cleaning procedure and before film formation (Fig. 1 (sample # 5, **5_FCA-N_0** [1]) and for successfully grown films (Fig. 2, **0**_**FCA_1** (non-modified alumina), Fig. 3 (modified sample # 5, **5_FCA-N_1**)). In Fig. 4 appearance of N1s signal and N1s line evolution during NO treatment (**6_FCA-N_1,** 7 × 10^−7^ mbar NO exposition at 670°C) are shown. The plots in Fig. 5, Fig. 6, Fig. 7 (**5_FCA-N_1)** confirms the reproducibility of angular dependences for N – doped films prepared by the proposed procedure. Figs. 8–11 shows evolution of Al2p, O1s, Cr2p and Fe2p lines during N-doped film formation (sample # 3, (**3_FCA-N_0**) and (**3_FCA-N_1**) [1]). Both segregation of aluminum and oxygen as well as reduction of FeOx and CrOx due to aluminothermy are well seen (Section 3.2 Mechanism of alumina film formation [1])*.* In Fig. 12 (**6_FCA-N_1**), XPS spectra of N1s line before and after NO treatment, when NO presents in residual vacuum, are shown in Fig. 13 (sample **Au_FCA-N**), the survey XPS spectrum shows presence of Au4f line after vacuum vapor deposition of the gold on N-doped AlOx/FeCrAl. Figs. 14 and 15 shows STM images of gold nanoparticles deposited by vacuum vapor deposition on non-modified AlOx/FeCrAl (Fig. 14, sample **Au_FCA**) and N-doped AlOx/FeCrAl (Fig. 15, sample **Au_FCA-N**) samples confirms absence of restrictions for thermal sintering of particles (small particle size (less than 3 nm) and short distance between particles).

In Table 1 see the list of samples mentioned in this article with brief description (sample numbering is the same as used in [1]).

**Table 1:** List of samples mentioned in this article.

Sample	Brief description
**0**_**FCA_1**	Sample # 0: non-modified AlOx film over FeCrAl substrate
**3_FCA-N_0**	Sample # 3: FeCrAl foil after Ar^+^ etching
**3_FCA-N_1**	Sample # 3: modified AlOx-N film over FeCrAl substrate prepared at 1.5 × 10^−7^ mbar of NO at 670°C
**5_FCA-N_0**	Sample # 5: FeCrAl foil after Ar^+^ etching
**5_FCA-N_1**	Sample # 5: modified AlOx-N film over FeCrAl substrate prepared at 6 × 10^−7^ mbar of NO at 670°C
**6_FCA-N_1**	Sample # 6: modified AlOx-N film over FeCrAl substrate prepared at 7 × 10^−7^ mbar of NO at 670°C
**Au_FCA-N**	Au nanoparticles deposited on N-doped AlOx/FeCrAl support
**Au_FCA**	Au nanoparticles deposited on non-modified AlOx/FeCrAl support

**XPS data**

Files in the data repository are organized in folders according to methods of sample's study: XPS (X-ray photoelectron spectroscopy) and STM (scanning tunneling microscopy). ‘XPS’ folder includes XPS spectrum data files (Excel files). Files are named according to the following convention ‘Fig X spectrum type sample name.xls’, where “X” is the number of the figure (when ‘Fig X-Y…’ file name is used, “Y” is the number of spectrum in figure); “spectrum type” is ‘survey spectrum’ or ‘region spectrum’ (regions are specified commonly for XPS); “sample name” is according to the Table 1. Every file includes description of the sample treatment when XPS spectrum was recorded; MgKa is used radiation; HV50 is HV used for spectrum recording; table with spectrum (two columns: BE (binding energy) in eV (electron-volt) and intensity in a.u. (arbitrary units). Tables can be used to plot spectrum using any appropriate software (for example, Excel). File Fig. 4 XPS N1s spectra 6_FCA-N_1.xls includes six spectra of nitrogen line at different NO exposition time (t = X s, where X is the exposition time in seconds). File Fig. 12 XPS N1s spectra 6_FCA-N_1.xls includes two spectra of nitrogen line before and after NO exposition. ‘STM’ folder includes STM images (JPEG files). Files are named according to the following convention ‘Fig. XX sample name.xls’, where “X” is the number of the figure; “sample name” is according to the Table 1. STM image size is 13.4 nm x 8.5 nm for Fig. 14 Au_FCA.jpg file and 24.0 nm x 14.6 nm for Fig. 15 Au_FCA-N.jpg file.

## 2 Experimental Design, Materials and Methods

The preparation of all samples is described in principle in “N–doping of alumina thin film support to improve the thermal stability of catalysts: preparation and investigation” [1]. Here we present the extended description of this procedure.

### 2.1 The choise of proper material

A foil of aluminum-containing heat-resistant steel Fecralloy® alloy (Goodfellow Cambridge Limited, England) in “as rolled” condition was used for the film preparation. The FeCrAlloy foil of this trademark was chosen as commercially available and inexpensive material. Use of such material as substrate allows reproducible preparation of unrestricted number of samples of supports and catalysts. According to the datasheet, the alloy composition is (wt. %): Fe – 72.8%, Cr – 22%, Al – 5%, Y – 0.1%, Zr – 0.1%. The thickness of the foil (1 mm) was chosen from the offered list to prevent bending of the samples and as result a damage of alumina film continuity during the further manipulations with sample (XPS to STM sample holder transfer and so on).

It is important to use **“as rolled”** rather than “annealed” foil condition since in this case the amount of dissolved oxygen is enough for formation of the initial alumina film over the surface of steel foil [1]. All the samples were shaped as disks with a diameter of ∼8 mm and a thickness of 1 mm to meet the dimensions of STM sample holder.

### 2.2 Cleaning protocol

Before the mounting on sample holder loading the samples were cleaned in ultrasound bath (96% ethanol) to remove technical contamination of steel rolling and shape machining.

Prior to the film preparation both sides of the specimens were cleaned by argon sputtering to remove surface contaminations as well as the major part of the surface native oxides. The regimes of argon sputtering (Ar pressure, current and duration) were chosen to achieve Fe/C atomic ratio of ∼5 as a criterion for the quality of cleaning. It should be mentioned that the comparable fractions of metal and oxide aluminum have to be well seen after argon sputtering (see Fig. 8). The purpose of this stage is to remove the mixed oxide barrier which prevents the future diffusion of aluminum to the surface of specimens.

### 2.3 Alumina film preparation

After cleaning procedure steel foil samples were used for preparation of N–doped alumina thin film support following procedure described in “N–doping of alumina thin film support to improve the thermal stability of catalysts: preparation and investigation” [1], based on sample annealing in vacuum at 670°C followed by NO treatment at 670°C and NO pressure in range of 1 × 10^−7^ mbar – 1 × 10^−6^ mbar.

**STM data**

**Fig. 16:**
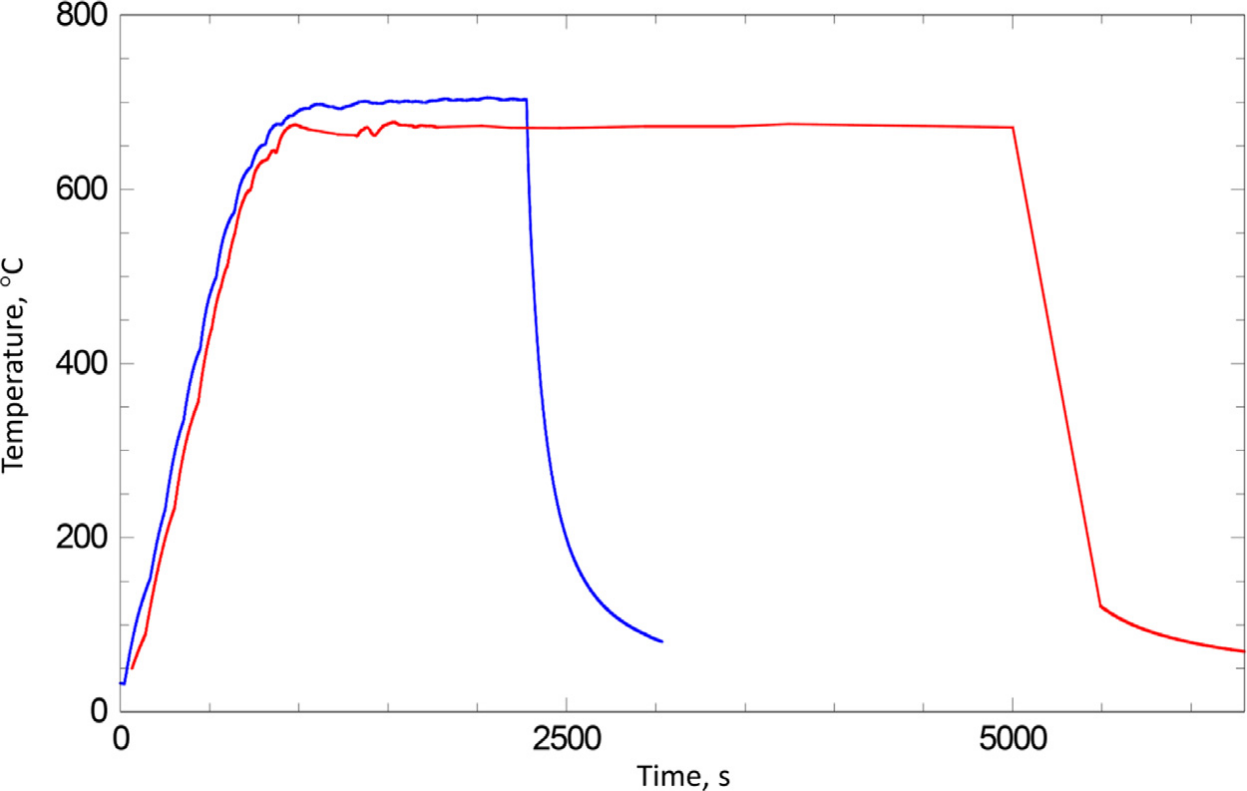
Typical temperature profiles for the pre-treatment heating up to 700°C (blue) and heating up to 670°C for film growth (red).

**Scheme 1:**
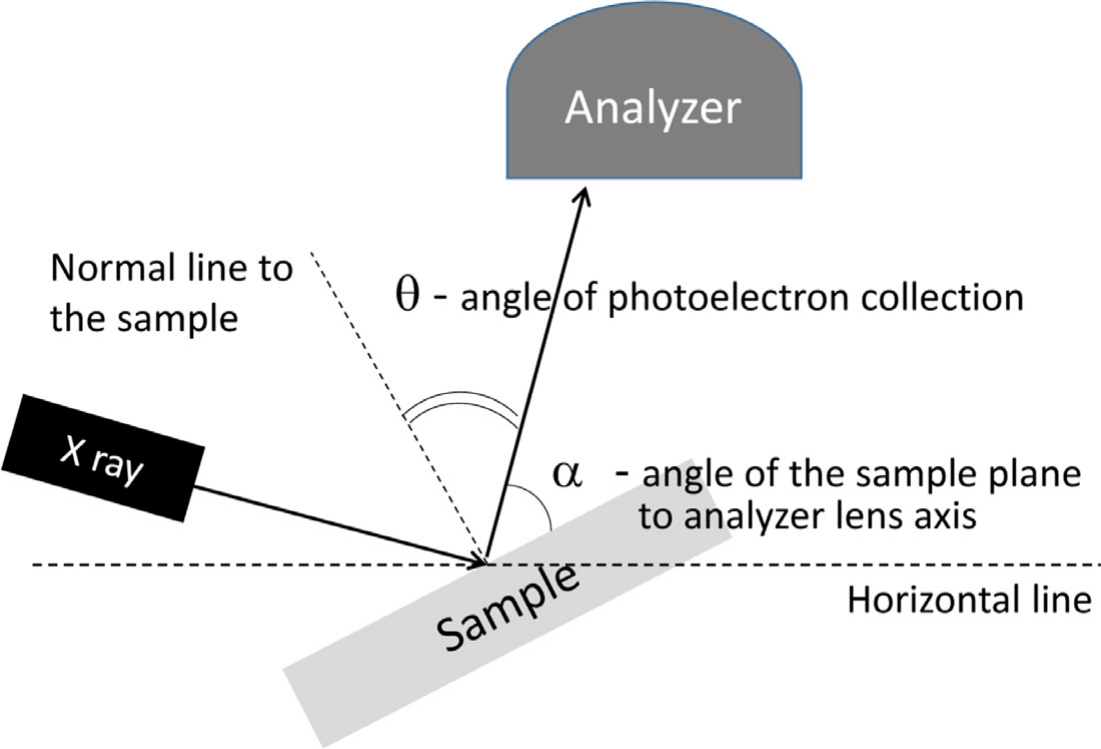
Angular resolved XPS experiments.

To use resistive heating to anneal the sample tungsten wire legs (∅ 0.2 mm) on tantalum springs were spot-welded to the backside of the foil disks. Tungsten legs on tantalum springs prevent the distortion of the sample position during the annealing, which is important for precise angle resolved XPS experiments (Scheme 1). The sample temperature was measured by a K type thermocouple spot-welded directly to the back side of samples. Standard water cooling of the XPS spectrometer sample holder was used to cool down the “cold” ends of thermocouples for precise temperature measuring. Resistive heating allowed fast ramping of the sample temperature (β = 1 C/s, see Fig. 16) as well as fast cooling (by turning off the power of power supply).

For all foil samples, a cycle of Ar^+^ sputtering followed by vacuum annealing at 700°C for 20 min and final Ar^+^ sputtering was used as pretreatment to remove possible contaminations with low melting point. After that all the subsequent procedures were carried out at temperature lower than 700°C (except for the temperature stability tests) and 670°C was pointed as the highest recommended temperature of using the samples [1].

The flows of NO and O_2_ were tuned up in a by-pass line by leak valves and then switched directly into an XPS analytic chamber to carry out *in situ* XPS experiments. This allowed controlling NO / O_2_ exposition precisely, which was important for the final film preparation, minimizing the time of NO / O_2_ flow stabilization when leak valve is used. The initial residual gas pressure in the chamber was ∼10^−9^ mbar, rising to 10^−8^ mbar between *in situ* experiments. The influence of NO presence in residual vacuum is shown in Fig. 12.

### 2.4 X-ray photoelectron spectroscopy

X-ray photoelectron spectroscopy study was performed at a VG ESCALAB HP electron spectrometer equipped with retardation grid in front of entry slit of hemispherical analyzer and single channeltron detector [3]. The nonmonochromatic Mg Kα line was used as the primary excitation. The spectrometer was calibrated using the Au4f_7/2_ (binding energy BE = 84.0 eV), Ag3d_5/2_ (368.3 eV) and Cu2p_3/2_ (932.7 eV) peaks [4]. The position of Fe2p line for metallic iron (BE = 707.0 eV) has been used as the internal standard for spectra calibration [5]. The binding energy values and the areas of XPS peaks were determined after Shirley background subtraction and analysis of line shapes using XPS peak software [6,7]. Sample holder allows precise adjustment of the angle of photoelectron collection, angle between the surface normal and the analyzer lens axis within the range of θ = 85° - 30°, for angular resolved XPS measurements (see Scheme 1).

### 2.5 Scanning tunneling microscopy

STM experiments were performed on an UHV (ultrahigh-vacuum) variable temperature STM VT-7000 (RHK Technology, USA). The cut Pt/Ir tips were used for STM measurements. The quality of the tips was tested periodically by achieving the atomic resolution on highly oriented pyrolytic graphite. Both topography and tunneling current images were acquired simultaneously to recognize the regions of the different electron states (film and particles) or to check surface non-uniformity. The STM images were analyzed with WSxM 5.0 Develop 3.3 software [8].

## CRediT authorship contribution statement

**Aleksey M. Dmitrachkov:** Investigation, Data curation. **Ren I. Kvon:** Investigation, Writing – review & editing. **Anna V. Nartova:** Conceptualization, Methodology, Investigation, Data curation, Writing – original draft.

## Declaration of Competing Interest

The authors declare that they have no known competing financial interests or personal relationships which have or could be perceived to have influenced the work reported in this article.
